# Moving somatic gene editing to the clinic: routes to market access and reimbursement in Europe

**DOI:** 10.1038/s41431-021-00877-y

**Published:** 2021-04-14

**Authors:** Tessel Rigter, David Klein, Stephanie S. Weinreich, Martina C. Cornel

**Affiliations:** 1 Amsterdam UMC, Vrije Universiteit Amsterdam, Clinical Genetics, Section Community Genetics, Amsterdam Public Health research institute, Amsterdam, the Netherlands; 2 Dutch Health Care Institute, Zorginstituut Nederland, Diemen, the Netherlands

**Keywords:** Economics, Translational research, Drug regulation

## Abstract

Somatic gene editing (SGE) holds great promise for making genetic therapy possible for many monogenic conditions very soon. Is our current system of European market authorization and reimbursement ready for the expected tsunami of gene therapies? At a recent workshop of the Netherlands ZonMw consortium on ethical, legal, and social implications of personalized medicine, we discussed the current possibilities for bringing new gene therapies to the clinic. In Europe, it is not yet clear whether the route via the European medicines agency as an advanced therapy medicinal product is the most appropriate for evaluation of highly personalized SGE applications, although this may optimally guarantee safety and effectiveness. Compassionate use may ensure faster access than the centralized procedure but does not stimulate the commercial development of products. Prescription to named patients may only provide adequate access for single patients. Temporary authorization of use may allow access to medication half a year before formal market authorization has been granted, but may also have large budget impacts. Magistral compounding under a hospital exemption may be an attractive solution for rare, tailor-made applications at an acceptable price. To approve local experimental use of a therapy on a case-by-case basis may be fast, but does not guarantee optimal safety, effectiveness, and broad implementation. We argue that alternative routes should be considered for products developed for a market of large groups of patients versus unique personalized treatments. A balance between scientific evidence for safety and effectiveness, affordability, and fast access may demand a range of alternative solutions.

## Introduction

In the past, monogenic disorders were considered incurable and few treatment options were available. The emergence of somatic gene therapy (SGT) has created new hopes for treatment and might enable life-saving cures [1–3]. While current gene therapies deliver an additional DNA product in the cell, recently developed gene-editing tools, such as clustered regularly interspaced short palindromic repeats–CRISPR-associated proteins (CRISPR-Cas), have the potential to truly repair disease-causing variants. Somatic gene editing (SGE) could theoretically be used to repair thousands of different variants in thousands of genes in a highly personalized way.

The clinical potential of genome editing depends on safety, effectiveness, and delivery, but also on navigating the road to market: legal regulation and authorization, distribution, availability, costs, and reimbursement. For different parts of the world, market authorization and availability of treatment are governed by different legislative systems, for example, the food and drug administration (FDA) in the USA and the European medicines agency (EMA) in the European Union (EU) [4]. These systems aim to regulate treatments as products, while for genome editing it may be needed to optimize the clinical procedure, both for ex vivo and in vivo editing. This would include the use of tailored guide-RNAs, certified laboratory equipment, and trained staff.

SGE does not neatly fall into any clear category for regulation by current legislative systems. Since monogenic disorders are rare, the number of patients needing specific treatments may be very small, and therefore the commercial development of a product may not be very attractive for drug manufacturers. Financial incentives already exist for orphan medicinal products [5]. However, due to the relatively small market, costly development, and market exclusivity, the price of some orphan drugs is still extremely high, limiting the affordability and thus available for patients.

Doudna et al. state that per-patient cost and fair access of gene therapies should be addressed not solely for application in patients with rare diseases, but also with consideration of the possibility that sometimes a standardized product may be suitable for a large, international population [1]. For example, a CRISPR-Cas product that is now under development for sickle cell disease and beta-thalassemia (CTX001) might serve a relatively large number of patients [6]. Would this CRISPR-Cas-based gene therapy become available globally in a fast and affordable manner?

Gupta strategists estimated that the average R&D costs per new molecular entity for an orphan disease could be around 0.5 bln USD [7]. More than 7% of total pharmaceutical expenditure in 2017 was for orphan medicinal products, and this percentage has not stopped increasing since [8]. This indicates a shift towards expenditure in higher cost, lower volume patient populations, and a shift in drug development towards more specialized targeting of diseases for higher unmet needs [8].

**Fig. 1:**
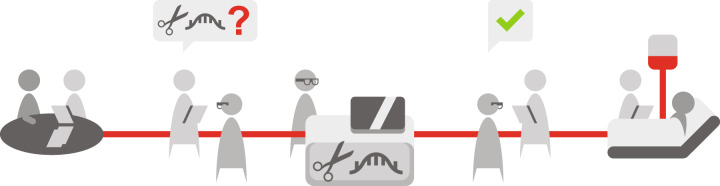
Flow of gene therapy from the clinical need to patient access. The flow emphasizes which aspects of quality control might need optimization. Aspects include the selection of tailored products (i.e., guide RNAs for the specific mutation of the patient in combination with a template for repair); certified laboratory equipment; and well-trained staff working according to clinical protocols.

EMA’s Committee for medicinal products for human use lists six advanced therapy medicinal products (ATMPs) on its list of applications for new human medicines for 2021 [9]. Clinicaltrials.gov, however, lists 4702 studies for “gene therapy” as of February 2021. The expected quick rise in applications for gene editing, in the face of possible patch-work regulation by member states and hurdles to accessing, leads to the following questions: is the current system of market authorization and reimbursement in Europe ready for the potential tsunami of gene therapies? How can patient access to gene therapy be ensured in the years to come? In brief, there is friction between the current legislative approach to gene therapy as a medicinal product and the clinical approach to gene therapy as a personalized procedure to cure the patient (Fig. 1).

The consortium ethical, legal, and social issues of personalized medicine (ELSI-PM) organized a workshop on the fifth of March 2020 to discuss potential ways forward. ELSI-PM is supported by ZonMw (the Netherlands Organization for Health Research and Development).

Using input from the workshop, we will briefly describe the current situation of market approval for SGT, including SGE, in Europe via the EMA. Next, we will discuss the pros and cons of potential alternative routes for patient access, market authorization, and reimbursement in the EU for SGE specifically, including a few examples of accelerated patient access to very innovative treatments. We follow the order starting with the regular European route to national to local solutions, ending with very personal (and therefore local) solutions.

## Market approval as ATMPs through the EMA

When a new therapy is being developed, several steps must be taken before patients can receive it as part of regular health care. Product developers typically submit a dossier to the EMA, which carries out a thorough assessment of the data including safety, efficacy, and legislative requirements before granting market authorization. Whereas on the one hand, this protects patients from adverse effects, on the other hand, the development and evaluation of the dossier takes significant time and resources and may delay patient access to effective drugs for unmet medical needs. In Europe, special legislation is in place for ATMPs. ATMPs benefit from a single evaluation and authorization procedure for all member states and include biological medicines such as gene therapy medicinal products (GTMPs) [10]. Note, however, that it has been questioned whether CRISPR-Cas therapies would be covered by European legislation for GTMPs, which are defined as consisting of “recombinant nucleic acid” since the DNA alterations in CRISPR-Cas9 gene therapy products are not produced by recombinant DNA technology [11].

Market approval is not only labor-intensive for both the pharmaceutical company and the regulatory agency, it can also come with specific requirements for the products after approval such as risk-mitigating measures. The EMA’s authorization of an ATMP is valid in the entire EU jurisdiction, suggesting broad accessibility. Yet, to ensure that the new technology is offered as high-quality care, it may be desirable to centralize expertise and implement it in a limited number of centers. For example, the market authorization of Yescarta™, a CAR T-cell product, stipulates that prior to the launch of the product in each member state, the company must make arrangements with the national competent authority about educational materials, training, and a guidance document for health care providers and an educational program for patients to explain the risks, such as cytokine storms, requiring that they remain in the proximity of the location where they received the infusion for at least 4 weeks [12]. At the same time, these extra risk-mitigating measures which require action at the national level effectively limit the number of centers where the new product can be administered. Besides the EMA’s stipulations, national reimbursement authorities may also want to limit the number of treatment centers in order to foster expertise [13]. Consensus on and organization of such a center of expertise might further extend the time it takes before patients have access to treatment. Furthermore, the price of some of these products is extremely high, limiting the affordability and thus available for patients. An example of an ATMP, designated as an orphan gene therapy product that was conditionally approved by EMA in 2020, is Zolgensma™. At $2.1 million, it is the most expensive drug worldwide [14, 15]. In general, annual treatment cost per non-oncological rare disease patient may be ~300 k€, much higher than often cited thresholds for cost-effectiveness, but the budgetary impact still is limited [16].

While CRISPR-Cas is “efficient, simple and cheap” according to some, a labor-intensive route for marketing authorization of orphan drugs for thousands of variants in thousands of genes may lead to unnecessary delays, and thus treatments may effectively not be affordable and accessible [14]. A summary of characteristics for the ATMP/EMA route and alternative routes to market access is provided in Table 1. The following sections will further elaborate on the pros and cons of the different alternative routes.

**Table 1:** Summary of characteristics for the routes to access to personalized medication.

Route	Example	Main authority	Initiator(s)	Level of approval	Funding	Main requirement	Benefit	Disadvantage
ATMP	Yescarta/ Luxturna [13]	EMA	Pharmaceutical company	European	National decisions after market access	The product should be defined as an ATMP and safety evaluated in a dossier	Systematic large-scale evaluation of safety and efficacy of the specific product, often with requirements after approval	Lengthy and labor-intensive, impacting pricing of drugs
Compassionate use	No SGTs registered	EMA advises national authorities	Physician attunes with the pharmaceutical company	National	Pharmaceutical company	Unmet medical need and clinical research or market approval procedures are ongoing	Access before market approval for (small) groups of patients with a serious unmet medical need	Temporary arrangement. The decreased incentive for commercial companies to invest in drugs for small groups of patients (no income during CU-phase) and selection of patients in a phase where limited stock is available is challenging (leading to international differences, or even lotteries) [18]
Named patients	Ephedrine for myasthenia gravis (not SGT) [29]	Dutch Health and Youth Care Inspectorate [19]	Doctors declaration required. Access granted through exemption at the individual level (permission for one patient)	National	reimbursement at the discretion of health insurance company [19]	The unmet medical need for an individual patient and availability of the product in another country, possibly for another indication [19]	Tailored access for the patient with unmet medical needs to treatments for very rare conditions	Permission is only granted on a case-to-case basis for one patient (labor-intensive/not suitable for large groups), differences in national regulations, not necessarily reimbursed (in the Netherlands). A supplying party is responsible for safety and follow-up [19]
Temporary authorization of use (French ATU)	Yescarta [30]	National medicines agency [31]	nATU: Requested by a physician (permission for one patient); cATU: Requested by drug manufacturer (permission for a (sub-)group of patients) [22]	National	French Ministry of Health and French National Health Insurance [20]	Urgent unmet medical need, presumed efficacy, and safety based on scientific data [31]	Access before market approval for patients with unmet medical needs, complete reimbursement, strong financial incentives for commercial companies [20]	Under construction, different maturity levels in different legislations, and potentially large budget impact for health care systems
IRB	Milasen, Fanconi [23, 24]	Local (national) research board	Researchers, often in collaboration with clinical experts	Local	Research funding	Local (national) criteria for safe and responsible research	Fast	Only small-scale application, not sustainable and equitable
Compounding	CDCA (not SGT) [32]	Hospital pharmacy; eventually National Health Care Institute [33]	Treating physician	Local	Reimbursement at the discretion of health insurance companies; eventually uptake in national health care package may occur	Production for individuals or very small groups of patients	Medicines can be produced at a lower price than a commercial product	Tension in regulatory framework (lack of precedents)
Hospital exemption	–	Health and Youth Care Inspectorate of Ministry of Health, Welfare and Sport [34]	Requested by pharmaceutical company or board of directors of a hospital	Local and national	Health insurance or hospital	ATMP’s	Potentially faster and cheaper than commercial production	Lack of transparency

## Compassionate use

In the period before a market authorization is granted, patients can sometimes get access to a product via a compassionate use program. The committee for medicinal products for human use of the EMA has the legal authority to advise national agencies to permit compassionate use programs, which are valid for one year. Compassionate use programs provide medicines to patients “free of costs”, for example after they enrolled in a clinical trial. This brings about a societal quid pro quo. The company is able to build a dossier for the EMA on the basis of a clinical trial. Afterward, patients who benefitted from the treatment retain access to the therapy, and the company benefits from publicity and “customer loyalty”, while the medicine is not yet generating income. Compassionate use designation might effectively bridge the time between a clinical trial and a product entering the market. However, it does not incentivize early or large-scale production and access.

At the Dutch ELSI-PM workshop, a particular problem for pharmaceutical companies was mentioned, namely that consistent manufacturing of large batches of the product cannot be guaranteed in this phase when the product is not yet generating income. Thus companies may have limited products available for patients outside clinical trials. Selecting countries for compassionate use programs and then selecting patients eligible for these products can be a challenge. Therefore, a company recently provided some doses for free to children with spinal muscular atrophy who were selected by lottery [17]. However, the perceived inequity is ethically problematic [18].

## Named patients (doctor’s declaration)

Like compassionate use, named-patient prescriptions, sometimes also referred to as doctor’s declaration, exist for medicines that are not marketed for the specific indication in the country. In the Netherlands, this route is often used for rare diseases, though not for ATMPs. There are two disadvantages: the permission is only valid for an individual, and medicines provided via doctor’s declaration are not necessarily reimbursed [19].

## Temporary authorization for use (ATU)

In France, the ATU system encompasses aspects of both compassionate use and named-patient use, but with a different system of reimbursement prior to market approval [20]. During the ATU validity period, products are entirely reimbursed by the French National Health Insurance, potentially having a large budget impact on the French healthcare system [21]. Manufacturers are required to pay back the total revenue difference between ATU pricing and the final negotiated price after market access in the case of a lower post-launch price. However, in the opposite case, manufacturers are compensated for every sold product to the level of the determined final product price [22]. For SGT, the ATU system may provide an incentive for early patient access to innovative medication during the market approval procedure.

## Local institutional review board approval

Some innovative gene therapies have been provided in a research setting after local institutional review board (IRB) approval. In Spain, for example, local IRB approval was given for the application of hematopoietic gene therapy for four Fanconi anemia patients [23]. Similarly, Kim et al. performed an “N-of-1” study for the development of milasen—a patient-customized oligonucleotide therapy—for the treatment of a life-threatening form of Batten’s disease [24]. Financing for the production of milasen was crowd-funded. This raises questions of equity and sustainability: will (the parents of) every patient with a rare disease be able to raise funding for his own treatment? Would society consider this funding model fair and just?

The IRB route has clear ties to the framework of research. On the one hand, the examples illustrate fast translation to patient care. On the other hand, this is not a sustainable solution to guarantee safety and effectiveness for all patients eligible for such treatments. In a large-scale clinical framework, it may not be feasible to assess effectiveness and safety per patient. Furthermore, the experience needed for such assessment may be better guaranteed by (inter)national agencies.

## Compounding under a hospital exemption

Compounding by (hospital) pharmacies has been historically used to tailor dose and form of otherwise standardized pharmaceutical products, e.g., if a patient cannot swallow a pill the pharmacist may provide the drug as a liquid. When novel expensive treatments such as SGE become available, a possibility may be to compound the treatment in hospital pharmacies at a significantly lower price. Recent legislation in the Netherlands has established that a compounded drug may also be reimbursed if it is equivalent to a registered product that was deemed to be effective but not cost-effective or if its budget impact is too high [25].

“Hospital exemption” is a similar pathway specific for treatments that meet the criteria for ATMPs. The ATMP must be produced on prescription and it must be made to order “for a specific patient”. In fact, production and dispensing for up to a maximum of five patients is considered production “for a specific patient”. The ATMP must be used under the exclusive professional responsibility of a physician. There are also procedural rules for the dispensing pharmacist. Permission to produce an ATMP under hospital exemption can only be granted if the ATMP is produced and used in the same country. A system for pharmacovigilance (drug safety) is required for all ATMPs, including those which are produced under hospital exemption. A disadvantage of the hospital exemption route is that there is currently a lack of overall transparency. If treatments are carried out under hospital exemption but outside the scope of clinical trials, the data are not publicly available. A group of European research universities has called for a voluntary, open-access registry of hospital exemption uses. This would facilitate access to strategies and data, provide evidence on (in)efficacy of treatments, and help identify which treatment-related costs need to be considered for reimbursement [26].

## Combining European regulation and local oversight: the Bio-Nespresso model?

Some gene editing with CRISPR-Cas may serve large patient numbers, and products may be brought to the market via the main EMA route for ATMPs. However, ultra-rare disease patients with rare gene variants may need personalized treatments, for which regimens similar to compounding and hospital exemption may be more suitable. A central question is how to ensure fast and equitable access while simultaneously guaranteeing optimal safety and effectiveness and reimbursement of costs of development and treatment.

Some medical treatments such as stem cell transplantation and surgery are regulated as health services, which require adequately trained personnel, monitoring, and evaluating results according to standard operational procedures. Quality control is organized at the level of the health service. CRISPR-Cas-based repair of specific disease-causing variants could be done in centers experienced in stem cell transplantation as well as proceedings related to CRISPR-Cas: drawing blood, isolating stem cells, modifying these cells ex vivo, and transfusing them back to the individual patient. The dispensing might resemble compounding. Such a model could circumvent many of the financial challenges of precision medicine. Also, bedside development under hospital exemption could be an affordable, safe and flexible regulatory context for CRISPR-Cas-based treatments. Could CRISPR-Cas as a technique be regulated as an ATMP, with individual products being prepared at the local hospital? Currently, this is not an option under EU Regulation 1394/2007.

A relevant analogy is the concept of a “Bio-Nespresso” machine for monoclonal antibodies, which do not fall under the ATMP regulation [27]. Its design goal is small-scale, timely production of monoclonal antibodies, according to the clinical needs of individual patients. It has been envisioned for the hospital pharmacy setting and would thus fall under the paradigm of compounding “which does not have to comply with the regulations commercially produced medicine have to” [27]. Similarly, if production-specific supplies are available in the hospital pharmacy or CRISPR-Cas facility, a tailored solution for every patient could be prepared at a “small-scale manufacturing unit for personalized medicine production” [27]. These therapies would have to be produced under good manufacturing practice (GMP) using standardized protocols that have to be adapted for each specific variant causing a specific genetic condition with controls and tests adapted to the individual patient and/or product. The system to guarantee the safety of these techniques at the CRISPR-facility remains to be developed. Novel oversight mechanisms are also needed for injections of guide-RNA, Cas-protein, and template, or if cells modified ex vivo by personalized CRISPR were given back to the patient.

## Towards coverage by basic health care insurance

Apart from having market access, SGE products also need to be financially accessible. Preferably, a funding model would also support the implementation of successful research findings into healthcare. As stated earlier, compassionate use is paid for by manufacturers. Named-patient use and compounded products may be covered by health insurers but at their discretion. Perhaps most relevant for CRISPR-Cas is an example of how commercial high-cost medicines are processed in the Netherlands. Most in-hospital treatments (including medicines) are automatically covered by the basic healthcare insurance package, without governmental assessment for relative effectiveness. But if a commercially produced medicine is at risk for high-budget impact of questionable cost-effectiveness, the Minister of Health can place it in the so-called “waiting room” or “sluice”. Such products can only be accepted into the insured package after assessment of its efficacy, and if the Ministry, after negotiations, can arrive at a reasonable financial arrangement with the supplier [28]. It is likely that commercially produced CRISPR-Cas products will be assessed in this way, but it is unclear whether and how compounded CRISPR-Cas treatments will be assessed for relative effectiveness. The guidance clearly also needs to be in place for compounding.

## Discussion

Different routes to market access can be considered for SGE therapies. In Europe, it is not yet clear whether the route via the EMA as an ATMP is the most appropriate, considering the highly personalized nature of most of the products, clinical procedures, and associated timetables, although this may optimally guarantee safety and effectiveness. SGE for thousands of specific variants involved in monogenic conditions requires a more flexible, personalized, and less labor-intensive route, especially if the patient groups are very small. Compassionate use may be fast for an individual patient, but it does not stimulate the commercial development of products. Prescription to named patients may only be a solution for single patients. Temporary authorization of use may allow access to medication half a year before formal market access has been granted, but it leads to financial uncertainty. Compounding under a hospital exemption may be an attractive solution for rare tailor-made applications at an acceptable price, but quality systems will need to be developed. Experimental therapy developed for research purposes and approved by local IRB is fast and adequate for small groups of patients but does not guarantee optimal safety, effectiveness, and broad implementation.

Perhaps a hybrid model of the existing routes is most appropriate to grant patients with very rare disorders relatively fast access to innovative SGE therapies while ensuring ensure safety and efficacy. It is conceivable that similar challenges and solutions may be relevant for innovative cancer drugs, making the development and implementation of new or adapted routes and attuning between stakeholders even more urgent. Economic aspects need to be considered to warrant affordable and equitable access.

## Conclusion

Gene therapies are becoming available at a rapid pace. Alternative routes to market access need to be considered, all with their own benefits and challenges. In order to realize safe, effective, and affordable therapies, scientists, clinicians, and bioethicists will need to collaborate with healthcare economists and regulators [1]. For CRISPR-Cas products that serve large patient populations, a route via EMA as ATMP may be most applicable, but for individualized therapies, regimens similar to compounding under a hospital exemption should be considered.

## Supplementary information

Tessel Rigter, PhD, explains in four minutes that very personalized medication such as somatic gene editing needs flexible routes to patient access, including a route for tailor made medication under local quality control next to the ATMP/EMA route.
